# Pupillary unrest, opioid intensity, and the impact of environmental stimulation on respiratory depression

**DOI:** 10.1007/s10877-021-00675-3

**Published:** 2021-03-02

**Authors:** Rachel Eshima McKay, Michael A. Kohn, Merlin D. Larson

**Affiliations:** 1 Department of Anesthesia and Perioperative Care, University of California San Francisco, San Francisco, CA USA; 2 Department of Epidemiology and Biostatistics, University of California San Francisco, San Francisco, CA USA; 3 Professor Emeritus, Department of Anesthesia and Perioperative Care, University of California San Francisco, San Francisco, CA USA

**Keywords:** Opioid induced respiratory depression, Opioid related patient safety, Infrared pupillometry, Monitoring drug effects, Opioid medication, pupillary responses, Opioid intoxication

## Abstract

Opioid-induced respiratory depression (OIRD) confers significant morbidity, but its onset can be challenging to recognize. Pain or stimulation effects of conversation may mask or attenuate common clinical manifestations of OIRD. We asked whether pupillary unrest could provide an objective signal of opioid exposure, and whether this signal would be independent from the confounding influence of extrinsic stimulation. We conducted a cross-over trial of healthy volunteers using identical remifentanil infusions separated by a washout period; in both, pupillary unrest in ambient light (PUAL) was measured at 2.5-min intervals. During one infusion, investigators continuously engaged the subject in conversation, while in the other, a quiet environment was maintained; measures of respiratory depression were compared under each condition. We tested PUAL’s relationship to estimated opioid concentration under quiet conditions, measured PUAL’s discrimination of lower versus higher opioid exposure using receiver operating characteristic (ROC) analysis, and assessed the effect of stimulation on PUAL versus opioid using mixed effects regression. Respiratory depression occurred more frequently under quiet conditions (p < 0.0001). Under both conditions, PUAL declined significantly over the course of the remifentanil infusion and rose during recovery (p < 0.0001). PUAL showed excellent discrimination in distinguishing higher versus absent-moderate opioid exposure (AUROC = 0.957 [0.929 to 0.985]), but was unaffected by interactive versus quiet conditions (mean difference, interactive – quiet = − 0.007, 95% CI − 0.016 to 0.002). PUAL is a consistent indicator of opioid effect, and distinguishes higher opioid concentrations independently of the stimulating effects of conversational interaction. Under equivalent opioid exposure, conversational interaction delayed the onset and minimized the severity of OIRD.

**Clinical trial registration:** NCT 04301895

## Introduction

Within the past decade, opioid-related adverse events have grown at unprecedented rates. Age-adjusted opioid overdoses have risen from 3.0 to 14.9 events per 100,000 people between 2000 and 2017, surpassing motor vehicles accidents and firearms as causes of accidental death in the United States [1]. Although the majority of overdoses occur in the community, cases among hospitalized patients continue to be reported, with iatrogenic respiratory arrest from opioid mismanagement cited as a significant source of preventable harm [2]. A recent administrative database reviewing hospital-related opioid-related cardiopulmonary arrest cases showed that approximately half of such incidents occurred in the intensive care unit, despite continuous assessment and monitoring [3]. Iatrogenic opioid-related respiratory depression (OIRD) carries severe liability; a closed claim analysis showed that among reported OIRD cases, > 75% produced death or serious brain injury, 1/3 occurred during continuous pulse oximetry monitoring, and 16% occurred within 15 min of an uneventful nursing check [4]. These reports suggest that OIRD can be difficult to recognize and predict. One favored approach currently under investigation involves remote monitoring systems to acquire and integrate vast quantities of patient data [5]. However, we ask whether nociceptive or conversational stimulation might obscure recognizable indicators of opioid toxicity, rendering common clinical parameters inadequately sensitive. We propose that an approach involving pupillary testing [6–8] may be more reliable than conventional measures in assessing opioid effect.

Under static levels of ambient light, the normal human eye exhibits continuous, bilaterally synchronous pupil size fluctuation [9, 10]. Within specific frequency bands, Fourier waveform analysis can transform these oscillations to a measure known as pupillary unrest in ambient light (PUAL) [11]. By convention, PUAL is expressed in arbitrary units (AU); additional details on the measurement appear in previous publications [11–14]. When an alert subject is in a dark environment [15], or undergoes general anesthesia [16], these oscillatory movements are abolished. Although its origins are unproven, PUAL appears to be mediated by fluctuating inhibitory activity within the parasympathetic Edinger Westphal nucleus, possibly driven indirectly by the locus coeruleus [17, 18]. Preliminary studies have suggested that PUAL declines after opioid administration, although the consistency and limits of this relationship have not been systematically established [11, 12, 14]. We argue that PUAL would be clinically useful if thresholds indicating clinically significant opioid exposure could be defined, but ask whether any such relationship might be minimized or superseded by environmental stimulation. To answer these questions, we performed a cross-over trial consisting of two identical opioid infusions separated by a washout period with contrasting levels of environmental stimulation. We hypothesized that: (1) respiratory depression would occur earlier and be more pronounced in the absence of stimulation; (2) PUAL would have a consistent inverse relationship to opioid concentration; and (3) the relationship between PUAL and opioid concentration would be unaffected by contrasting experimental conditions.

## Materials and methods

After receiving approval from the UCSF Institutional Review Board, we recruited 20 healthy volunteers aged 18–40 to participate in a crossover study consisting of two sequential 35-min remifentanil infusion regimens, identical except that in one, interactive conditions were maintained while investigators continuously engaged the subject in conversation, while in the other, quiet conditions were strictly maintained. A 30-min washout period separated each protocol, and the sequence (interactive-then-quiet versus quiet-then-interactive) alternated with each successive enrollee. Exclusion criteria included use of opioid agonist or antagonist within the prior 30 days, any cardiopulmonary or neurologic condition, diabetes, obstructive sleep apnea (OSA), BMI > 35 kg/m^2^, and current or previous substance use disorder. After an 8-h fasting period, subjects arrived at the UCSF Department of Anesthesia Hypoxia Lab, equipped with standard resuscitative equipment and medications. Lighting conditions (200 lx) were strictly controlled, and the room was free of distracting noise. After providing written informed consent, subjects received one peripheral IV, baseline pupillary and vital-sign measurements, and prophylactic antiemetic medication (aprepitant 40 mg + ondansetron 4 mg).

### Measurements

Pupillary measurements were obtained with a hand-held infrared pupillometer (Neuroptics PLR-3000, Laguna Hills, California), with each subject looking into a black rubber cone-shaped eye piece with the left eye. This eye piece was situated to exclude ambient light, while the operator’s left hand covering the contralateral eye. Since the pupil diameter does not fluctuate in darkness, a soft blurred disk of white light from a 50 µ-watt source, at approximately 350 lx illumination, was directed at the measured eye to initiate the oscillation in pupil size, and thereafter a 10-s infrared video of the pupil was taken. The videos were processed post hoc fast Fourier transformation to quantitate the PUAL measurement. Previous calibration of the PUAL, obtained by measuring metal holes of known diameter (2.6–4.8 mm), allowed subtraction of inherent noise and establishment of zero at the lower scale boundary [11]. In addition to PUAL, the average pupil diameter (millimeters) was recorded.

### Study protocol

In each 35-min test sequence, vital signs were continuously monitored and pupillary measurements were taken every 2.5 min. During the first 10 min of the 35-min sequence, remifentanil was infused at a predetermined rate described below. Under interactive conditions, sedation assessment was made using the Pasero Opioid-Induced Sedation (POSS) Scale [19].

### Opioid infusion

The remifentanil was infused for 10 min- at a rate of 0.2 µg/kg/min for the first 5 min, followed by 0.3 µg/kg/min for the next 5 min. After remifentanil discontinuation, pupillary measurements continued every 2.5 min for the remaining 25-min recovery phase. To avoid the stimulating effect of sequential blood drawing or the added risk of arterial line placement to facilitate repeated blood sampling, we did not measure remifentanil blood concentration, choosing instead to use an infusion protocol based on the Minto model that, when given to eligible subjects, would achieve an estimated maximum effect site concentration of 4–6 ng/mL, a level known to produce near-maximum isoflurane MAC reduction and high probability of apnea [20–22]. During both interactive and quiet conditions, the investigators avoided prompting the subjects to breathe, until and unless SpO_2_ fell to 90%. The recovery and washout periods between the two infusions were chosen so that in each case, subjects would start the second run of the two experiments at near-zero remifentanil concentration, and could realistically complete participation in the study within a 3-h window [21–23].

### Outcomes

Primary outcomes were (1) frequency of CO_2_ increase and desaturation in the quiet versus interactive conditions; (2) correlation between PUAL and intensity of opioid exposure (represented by time-points corresponding to progressively increasing and declining estimated opioid concentrations); and (3) impact of the quiet versus interactive conditions on opioid-related changes in PUAL.

### Sample size calculation

Assuming an average (SD) baseline PUAL of 0.246 (0.125) based on an observational sample of deidentified patients [14], we calculated that 17 subjects would provide 80% power to demonstrate a 50% decline in PUAL at 5 min, with an two-sided alpha = 0.05. We therefore we opted to enroll 20 subjects total.

### Statistical analyses

We tested 20 subjects under paired conditions- absent versus uninterrupted conversational interaction- over a 35-min period. After testing whether the sequence of conditions affected any outcomes, and observing no significant difference, all analyses were conducted disregarding the sequence in which the subjects experienced the background conditions.

#### Relationship between environmental condition (interactive versus quiet) and respiratory outcomes

To establish whether conversational interaction mitigated OIRD, we compared quiet versus interactive conditions in each subject on the following binary outcomes (McNemar’s test, 2 tails): oxyhemoglobin desaturation (SpO_2_ ≤ 90%), elevated transcutaneous CO_2_ (defined as ≥ 15% increase in above baseline), and ventilatory rate < 10/min. We compared the highest observed CO_2_ in each subject under each condition, and the maximum proportional increase in CO_2_ compared to baseline by the Wilcoxon signed rank test. Finally, we compared the time to onset of desaturation (SpO_2_ < 90%) in each subject under the two conditions using a conditional Cox proportional hazards model.

#### Correlation between PUAL and opioid concentration

We examined PUAL and opioid concentration under quiet conditions, during both drug infusion (0–10 min, where opioid concentrations progressively increased) and recovery (10–35 min, where opioid concentrations progressively declined). Each 2.5-min point was treated as an ordinal variable “time”, and opioid effect-site concentrations were calculated at each time point according to the Minto pharmacokinetic model estimates [22, 23]. The effect of time (as surrogate for opioid concentration) on PUAL was assessed using a generalized estimating equations (GEE) regression.

To test PUAL’s discrimination between high versus absent-to-moderate opioid exposure we used receiver operator characteristic (ROC) analysis, with 0 and 2.5 min time-points corresponding with absent-to-moderate opioid exposure, versus 5.0, 7.5, 10.0, and 12.5-min time points corresponding to high opioid exposure. Initially this approach was based on relative changes in estimated remifentanil effect site concentrations, but after completion of data collection we performed further analysis to confirm the validity of the approach, using both age and body-size characteristics of the participants and the average onset of desaturation.

#### Relationship between pupillary findings (PUAL and pupil diameter) and environmental stimulation

After establishing the relationship between PUAL and opioid exposure under quiet conditions (second objective), we compared PUAL under interactive versus quiet conditions using mixed effects regression, with experimental condition and timepoint as the categorical fixed effects, and subject number as the (categorical) random effect. The model outcome estimated the overall difference in PUAL in the two conditions (interactive – quiet). We also performed conditional Cox regression to compare the time from start of the remifentanil infusion until 90% PUAL suppression was reached under both quiet and interactive conditions.

The study was registered at clinicaltrials.gov on March 10, 2020 (NCT 04301895).

All analysis was performed using Stata 16 (College Station, TX).

## Results

Baseline PUAL ranged from 0.12 to 0.54 AU (median 0.27, IQR 0.18 to 0.33). Comparison of pre-infusion PUAL between Run 1 and Run 2 showed no meaningful difference (0.010; 95% CI − 0.026 to 0.045; p = 0.5870, Table 1).

**Table 1:** Study participant characteristics are listed below

Characteristics of study participants	
Age (years), mean (SD)	25.6 (3.3)
Sex (F/M), n	13/7
Height (cm), mean (SD)	166.8 (9.6)
Weight (kg), mean (SD)	64.7 (12.2)
BMI (kg/m^2^), mean (SD)	23.2 (4.1)
Baseline PUAL Run 1, mean (SD)	0.269 (.106)
Baseline PUAL Run 2, mean (SD)	0.260 (.103)

Paired t-test comparison of baseline PUAL at the beginning of Run 1 and Run 2: difference 0.010 (95% CI − 0.026 to 0.045; p = 0.5870)

### Respiratory outcomes under quiet versus interactive conditions

**Fig. 1:**
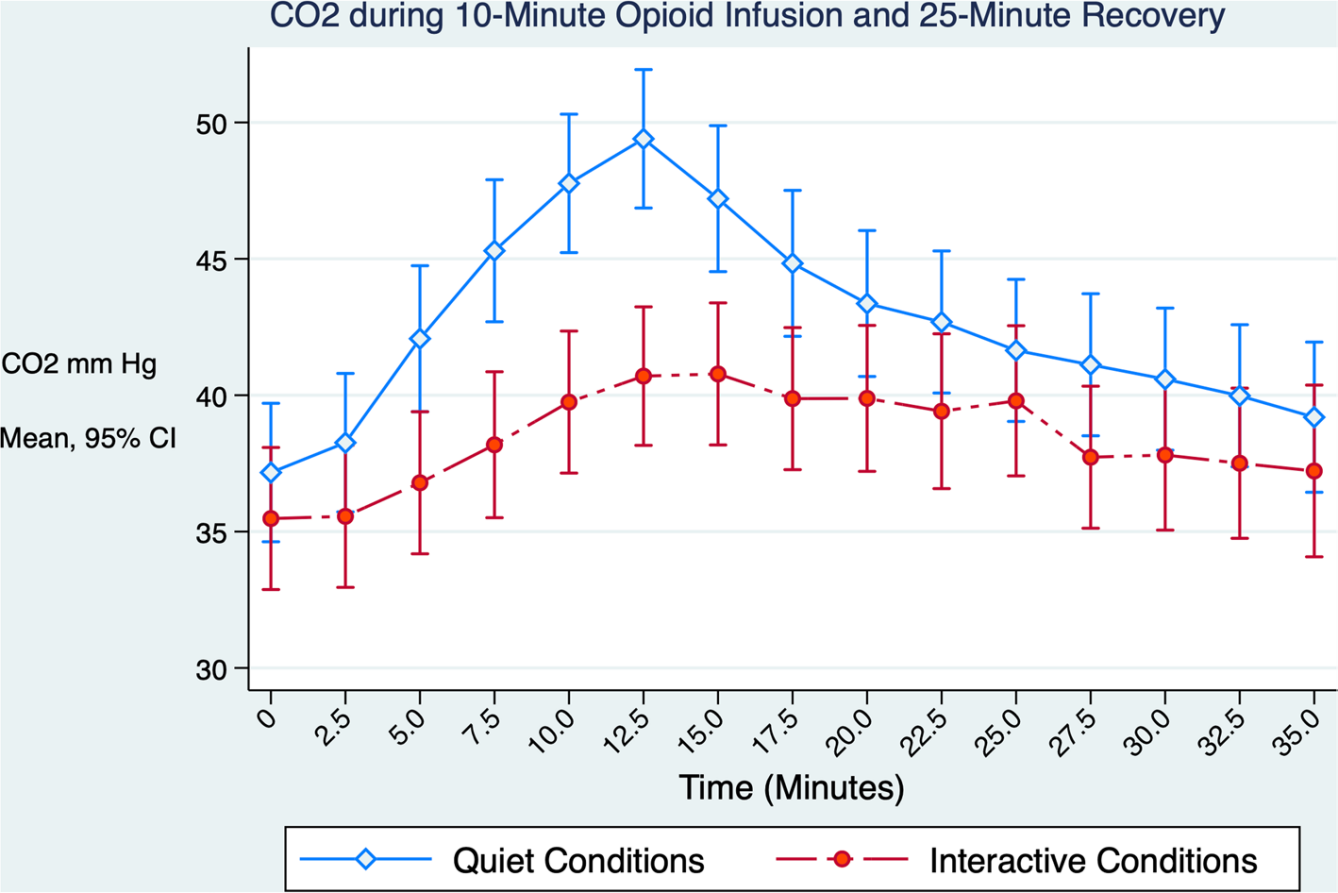
Mean transcutaneous CO_2_ measurements during the 10-min remifentanil infusion and 25-min recovery period. Respiratory depression was more pronounced during the quiet versus interactive conditions, with CO_2_ increasing 37% versus 21% above baseline values respectively (p = 0.0002)

**Fig. 2:**
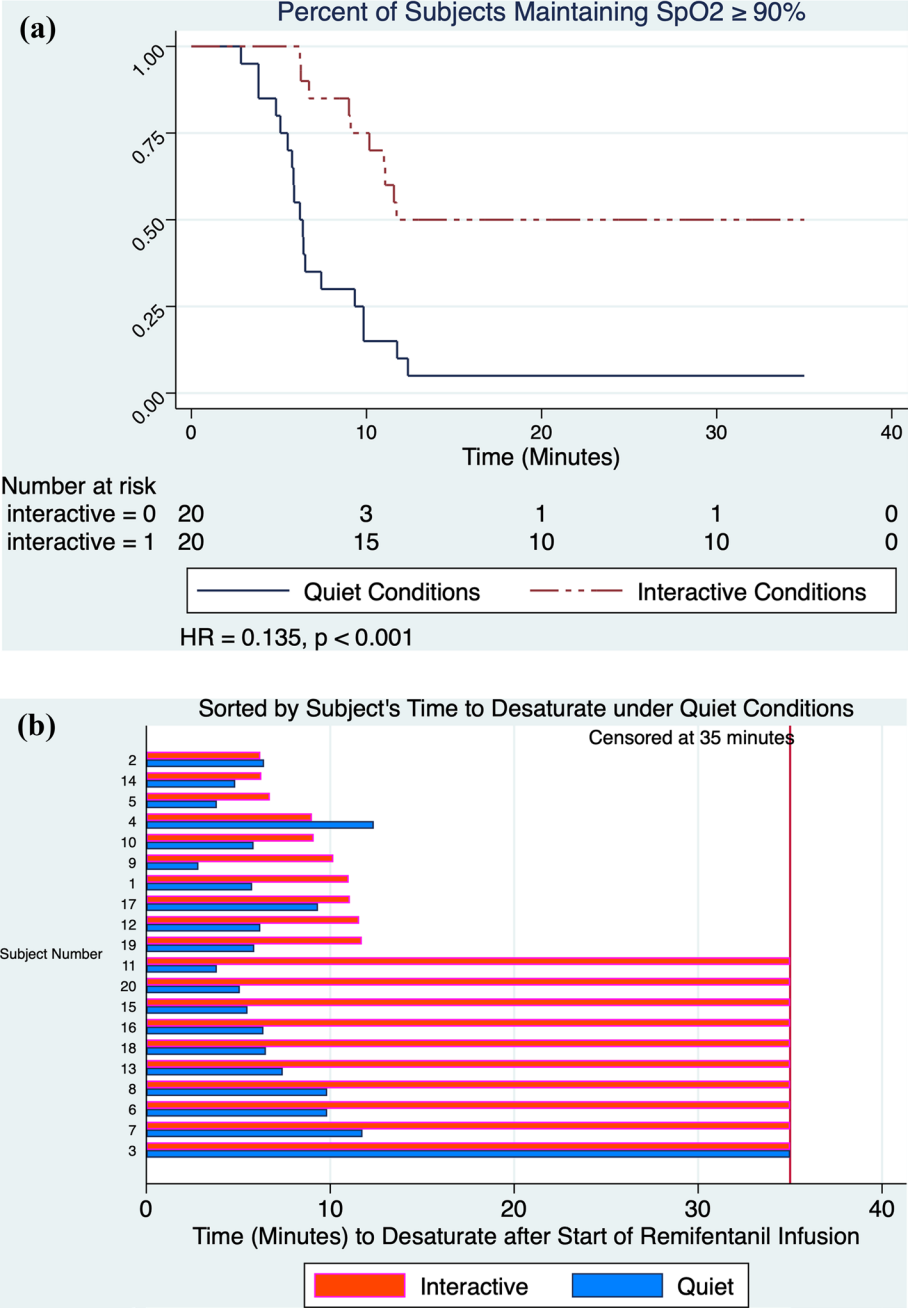
**a** Oxyhemoglobin desaturation occurred more frequently (in 19/20 in versus 10/20 subjects) and earlier (median onset 6.2 versus 9.6 min) under quiet compared to interactive conditions (HR 0.135, p < 0.001, conditional Cox regression). In contrast to respiratory outcomes, PUAL decline did not differ under quiet versus interactive conditions. **b** Paired data shows onset of oxyhemoglobin desaturation in subjects under quiet and interactive conditions

As the remifentanil infusion progressed, signs of OIRD were more frequent and pronounced under quiet versus interactive conditions. CO_2_ increased ≥ 15% above baseline in 20/20 versus 14/20 subjects (McNemar Exact p = 0.0312), ventilatory rate fell below 10 breaths per minute in 18/20 versus 5/20 subjects (McNemar Exact p = 0.0002, Table 2), the highest observed CO_2_ was significantly greater (50.8 versus 43.4 mm Hg, Wilcoxon signed-rank test p < 0.0001, Fig. 1), and the proportional CO_2_ increase above baseline was higher (37.5% versus 21.3% p = 0.0002, Table 2) compared to interactive conditions. Oxyhemoglobin desaturation occurred more often during quiet versus interactive conditions (in 19/20 versus 10/20 subjects, McNemar Exact p = 0.0039), and Conditional Cox regression confirmed that under interactive conditions, desaturation occurred at later time points (median time to desaturate = 9.6 [6.4–11.4] versus 6.2 [5.4–8.0] min, hazard ratio = 0.135 [0.054–0.339], p < 0.001, Fig. 2a and b).

**Table 2:** Summary of respiratory outcomes stratified by interactive versus quiet condition

Respiratory outcomes stratified by background experimental condition					
Measurement	Background condition				p-value^*^
	Interactive		Quiet		
	n	(%)	n	(%)	
Oxyhemoglobin desaturation	10	(50.0)	19	(95.0)	0.0039
Respiratory depression (≥ 15% CO_2_ increase)	14	(70.0)	20	(100)	0.0312
Respiratory rate < 10/min	5	(25.0)	18	(90.0)	0.0002

	Mean	(SD)	Mean	(SD)	p-value^**^
Highest observed CO_2_, median (IQR)	43.4	(40.7, 46.1)	50.8	(47.6, 54.0)	< 0.001
Highest proportional increase in CO_2_ above baseline (%)	21.3	(13.2)	37.5	(10.7)	0.0002

*p-value calculated using two-tailed McNemar’s exact test unless otherwise indicated

**p-value calculated using Wilcoxon signed rank test

### Relationship between PUAL and time (representing progressive changes in estimated opioid concentration)

**Fig. 3:**
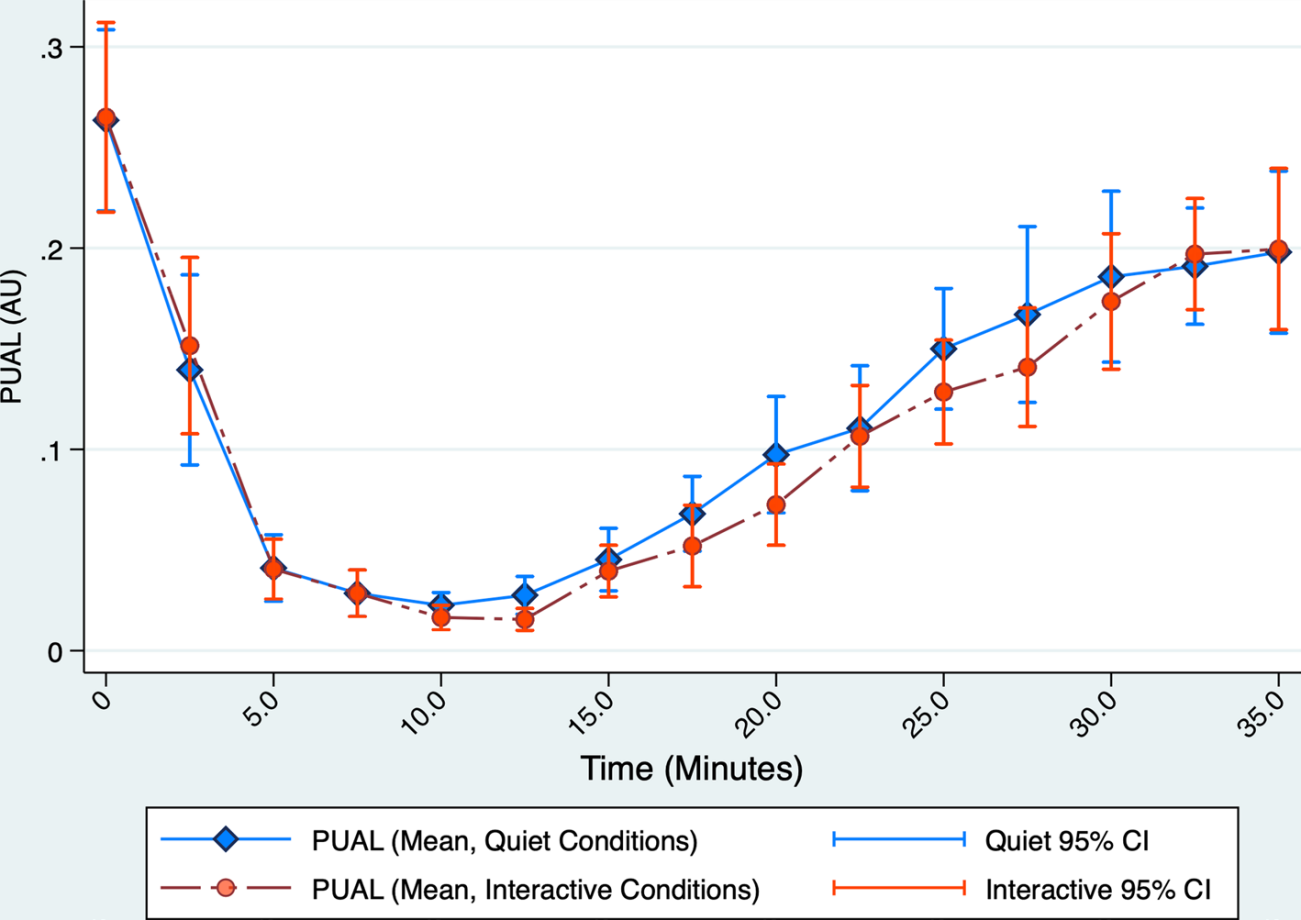
PUAL declined progressively as opioid concentration increased during the 10-min remifentanil infusion, from an average of 0.264 at baseline to 0.022 by 10 min under quiet conditions, and recovered as the infusion was discontinued (p < 0.001). The relationship between PUAL and opioid exposure did not differ significantly under interactive versus quiet conditions, with mean PUAL difference (interactive – quiet) = − 0.007 (− 0.016 to 0.019), p = 0.1240

During the remifentanil infusion (min 0–10), PUAL declined significantly at each 2.5-min juncture as remifentanil concentration incrased; under quiet conditions, from an average of 0.264 at baseline to 0.022 by 10-min (p < 0.001, Table 3 and Fig. 3).

**Table 3:** Summary of pupillary findings, under interactive and quiet conditions

PUAL and pupil diameter measurements under interactive and quiet conditions					
Measurement	Interactive		Quiet		p-value^*^
	Mean	(SD)	Mean	(SD)	
Pupillary unrest (PUAL)					
Baseline PUAL (AU)	0.265	(0.107)	0.263	(0.102)	0.987
PUAL (AU) after 5 min of infusion	0.040	(0.034)	0.041	(0.037)	0.930
PUAL (AU) after 10 min of infusion	0.016	(0.014)	0.022	(0.014)	0.183
Lowest PUAL (AU) during experiment	0.008	(0.010)	0.010	(0.008)	0.542
Percent PUAL decline at 5 min	82.0	(16.0)	81.2	(18.4)	0.974
Percent PUAL decline at 10 min	93.3	(6.0)	90.0	(9.2)	0.187
Maximum percent PUAL decline	96.4	(5.0)	95.8	(4.0)	0.678
Pupil diameter					
Baseline diameter (mm)	4.4	(0.5)	4.3	(0.6)	0.571
Diameter (mm) at 5 min of infusion	2.5	(0.4)	2.5	(0.4)	1.000
Diameter (mm) at 10 min of infusion	2.2	(0.2)	2.2	(0.2)	1.000
Lowest diameter during experiment	2.2	(0.2)	2.1	(0.2)	0.122
Percent diameter decline at 5 min	42.5	(9.5)	41.0	(10.4)	0.692
Percent diameter decline at 10 min	49.9	(6.6)	48.1	(6.6)	0.394
Maximum percent diameter decline	50.5	(6.2)	49.3	(6.9)	0.566

*p-values were calculated by paired t-test. PUAL and pupillary diameter measurements at 5-min and 10-min after the start of the remifentanil infusion and at maximum parameter decline all differed significantly from their respective baseline values, p < 0.0001

Pupil diameter likewise showed significant decline during these time intervals, but by a smaller percentage compared to PUAL (49.9% ± 6.4% diameter decline versus 95.1% ± 4.2% PUAL decline, t test p < 0.0001, Table 3).

**Fig. 4:**
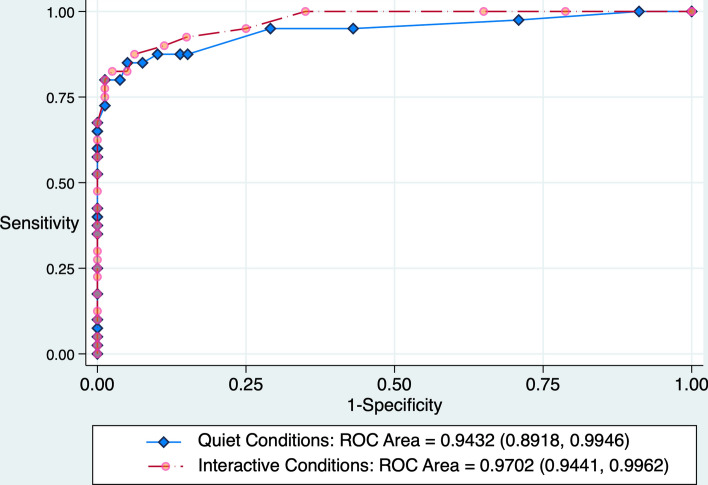
In 20 subjects under quiet conditions, PUAL showed excellent discrimination between high versus absent-moderate opioid effect: AUROC = 0.9459 (0.8957—0.9961) under quiet conditions and 0.9671 (0.9384–0.9958) under interactive conditions (p = 0.3588 for difference in ROC area under the two conditions). Compared to PUAL, CO_2_ showed weaker discrimination between high versus absent-moderate opioid effect: AUROC = 0.8079 (0.7284–0.8874) under quiet conditions and 0.6501 (0.5480–0.7521) under interactive conditions (p = 0.0202 for difference in CO_2_ ROC area under the two contrasting conditions, p = 0.0034 for difference between CO_2_ versus PUAL ROC area under quiet conditions, and p < 0.0001 for difference between CO_2_ versus PUAL ROC area under interactive conditions)

PUAL discriminated well between high opioid effect (time points 5.0–12.5 min) and zero to moderate opioid effect (time points 0 and 2.5 min), with AUROC of 0.9459 (0.8957–0.9961) in the 20 quiet experiments and 0.9671 (0.9384–0.9958) in the 20 interactive experiments (Fig. 4). PUAL values ranging from 0.00 to 0.04 were associated with an interval likelihood ratio = 14.6 (5.59 to 38.10) for high-dose opioid exposure, whereas values ≥ 0.13 were associated with an interval likelihood ratio = 0.017 (0.004 to 0.069). PUAL values > 0.04 but < 0.13 were indeterminant (LR = 1.15, 0.706 to 1.861). Adding further support to associating 5.0 to 12.5-min measurements with high opioid concentration, we noted that 26/29 (90%) of all desaturation events occurred between 5.0 and 12.5 min, while three occurred between 2.5 and 5.0 min.

Compared to PUAL, CO_2_ was significantly weaker in identifying absent-moderate versus high opioid exposure, and the discrimination was influenced by level of stimulation. AUROC for CO_2_ was 0.8079 (0.7284) during quiet conditions versus 0.6501 (0.5480–0.7521) under interactive conditions (p = 0.0202 for difference). By contrast, PUAL’s discrimination did not differ significantly under either condition (p = 0.3588).

**Fig. 5:**
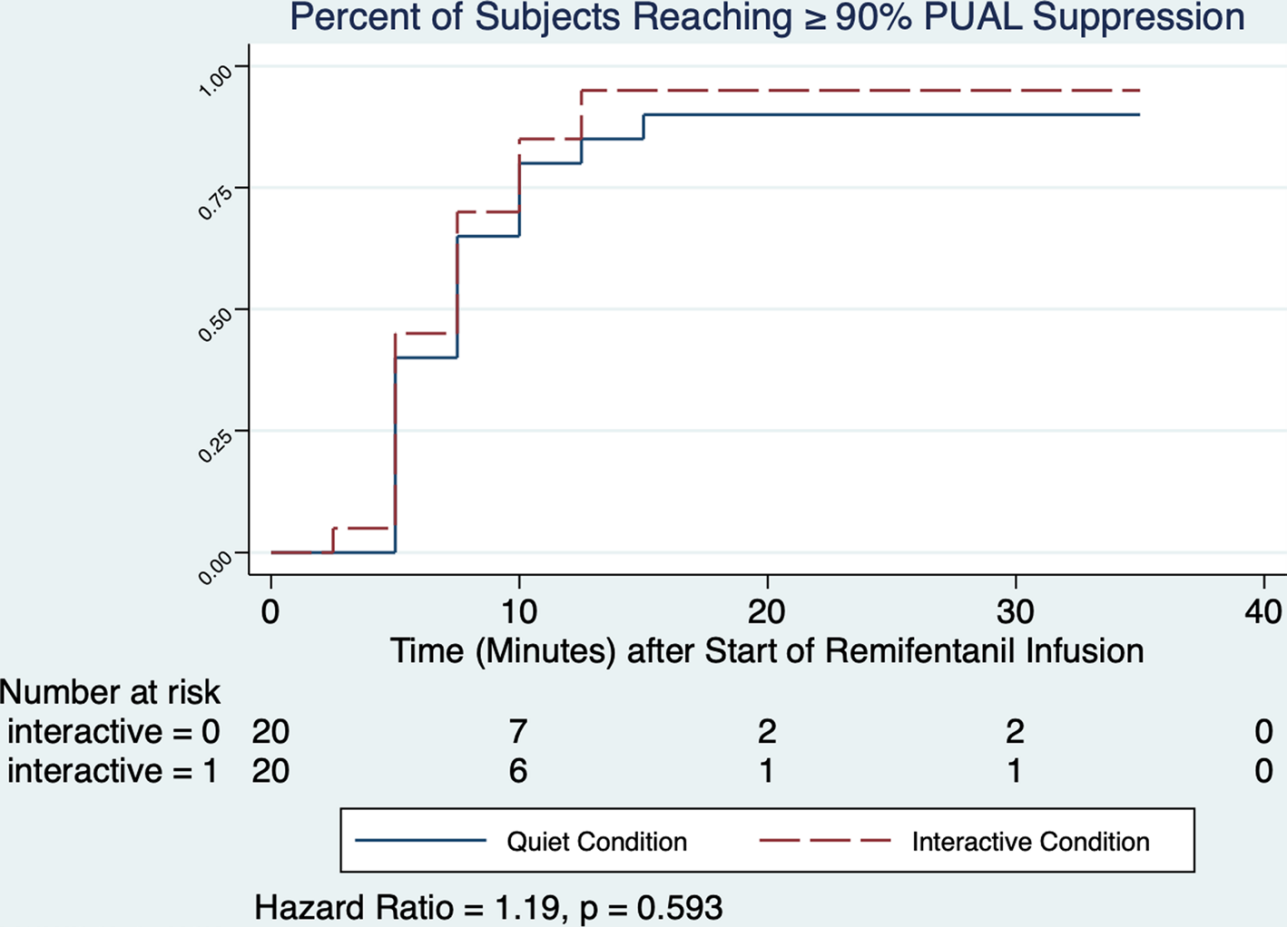
Overall, ≥ 90% PUAL suppression occurred in 19/20 of subjects during quiet conditions versus 18/20 subjects during interactive conditions. The proportion of subjects reaching ≥ 90% PUAL suppression under each condition did not differ significantly, HR 1.193 (95% CI 0.624–2.278), p = 0.593

PUAL suppression by 90% occurred in 18/20 quiet versus 19/20 interactive experiments (hazard ratio = 1.193 [0.625–2.278], p = 0.593, Fig. 5); average overall decline was 90(9)% at 10 min and 96(4)% at maximum suppression (Table 3). Mixed Effects regression showed a mean difference (interactive – quiet) under contrasting conditions of − 0.007 (− 0.016 to 0.019, p = 0.1240) during the 35-min experiment.

## Discussion

Volunteers receiving remifentanil experienced greater respiratory depression and more frequent oxyhemoglobin desaturation under quiet versus interactive conditions. Increasing opioid exposure was significantly and dose-dependently correlated to decline in PUAL. In contrast to respiratory outcomes, PUAL decline concurrent with progressive remifentanil infusion did not differ under interactive versus quiet conditions.

Midbrain-level evidence of opioid activity was demonstrated by the consistent obliteration and subsequent recovery of PUAL. The delayed, less frequent desaturation in the interactive setting supports the notion that cortical activity augments respiratory drive. In clinical settings, such forms of stimuli may include pain or periodic interaction with caregivers. Patients with persistent pain despite extensive opioid treatment who undergo respiratory arrest after receiving rescue neuraxial or peripheral nerve block with local anesthetic constitute one example [14, 24, 25]. Opioid-treated hospitalized patients exhibiting normal appearance and physiologic parameters who, when left alone, undergo respiratory arrest without receiving additional opioid constitute another example [14, 26].

OIRD is difficult to anticipate on the basis of formulas [27], and published cases of ICU-associated OIRD suggest that conventional clinical parameters are collectively insensitive as OIRD signals [2–4]. POSS scores performed poorly in our protocol; of the 10/20 interactive subjects with desaturation, 8/10 had POSS scores of 1 (lowest in the Likert Scale) throughout the infusion and recovery periods. Continuous pulse oximetry (CPO) is frequently advocated for hospitalized patients when concern for risk of OIRD is raised. When SpO_2_ readings fell to 90% in our experiments we immediately raised the FiO_2_; in all cases, the increased FiO_2_ caused SpO_2_ to rise and remain at acceptable levels (≥ 92%). However, that SpO_2_ decline from clinically acceptable (95–100%) to hypoxic ranges occurred within seconds. In a CPO unit, such an abrupt transition would require an intervention response-time on the order of seconds to prevent profound hypoxia; outside of an operating room, this response-time would seem unrealistic. The prompt SpO_2_ correction with modest increase in FiO_2_ underscores the fact that OIRD may go unrecognized in clinical settings during use of supplemental oxygen if detection relies on SpO_2_. Continuous capnography has also been proposed as a measure to detect OIRD, and although CO_2_ correlated with increasing opioid concentration and probability of desaturation, conversation blunted the relationship, and a broad range of measurements were observed coinciding with desaturation. Although respiratory rate < 10/min occurred more frequently in subjects experiencing desaturation, this threshold was less sensitive than ≥ 15% CO_2_ increase as a predictor. With rising CO_2_ and opioid exposure, an irregular pattern of ventilation as opposed to simple decline in rate, was observed, underscoring the limited sensitivity of respiratory rate in lieu of CO_2_ to indicate OIRD.

In contrast to conventional measures, pupillary measures were highly sensitive to opioid increase and onset of OIRD. While pupil diameter decline was highly correlated with increasing estimated opioid concentrations, PUAL became nearly obliterated as opioid concentration increased. Although both measures were sensitive, PUAL’s utility as a clinical marker is arguably greater. Diameter is an interval measurement, lacking an unambiguous, lower-limiting value. PUAL not only has a greater effect size, but also a ratio scale that includes a definitive lower-limiting value [28].

What are the practical implications of PUAL in clinical settings? Although the measurement is not a substitute for global clinical assessment, it could provide specific information and decision-support to the clinician when administering opioids to patients for treatment of acute or chronic pain. In hospitalized patients, the 10-s pupil scan for PUAL could be obtained at the time of admission, at subsequent intervals when standard vital signs are measured, and at specific junctures including introduction of concomitant depressant medication, intensification of analgesic treatment, or onset of altered mental status. In the ambulatory setting, PUAL could be useful to identify patients who may be impaired or at risk for OIRD when returning to their home environment. Low PUAL measurements may warrant a variety of responses depending on the clinical circumstances, including use of supplemental oxygen, implementation of continuous cardiorespiratory monitoring, opioid de-escalation, or transition to effective nonopioid analgesic strategies. Conversely, PUAL measurements above the low-risk threshold would indicate low likelihood of imminent, clinically significant OIRD, and would favor the safety of continuing opioid treatment if clinically indicated. Obtaining the measurement requires only simple training; however, interpretation of PUAL in a broad spectrum of clinical conditions will require clinical judgment and additional studies.

Our data confirm that OIRD can be partially antagonized by environmental factors that activate neocortical pathways. While active engagement with an opioid-medicated subject may enhance breathing, the impact of opioids on PUAL cannot be overcome by the same interaction. An explanation for this paradoxical effect has not yet been determined, although it is unlikely to be answered by laboratory studies because of the wide variety of opioid-related pupillary responses in experimental animals [29]. We theorized that in the presence of high-dose opioid, differential block of pathways contributing to maintenance of ventilatory drive would be observed. In the brainstem pathway, reflected by blockade of inhibitory influences of the EW nucleus [30] and PUAL suppression [11], the antagonism is consistent and highly correlated with opioid exposure. Conversely, in cortical pathways, influenced by behavioral interaction, activity remains partially intact as long as subjects remain conscious. These concepts are consistent with findings showing that under increasing propofol hypnosis, PUAL values remain within normal ranges until a subject becomes completely unresponsive [16]. Although activity in several brain centers has been associated with pupillary responses, the final inhibitory pathway in the EW nucleus that is blocked by opioids is not known [18].

There are several limitations to our study. First, opioid concentrations were not measured. Instead we relied on modelled concentrations to determine relative change in opioid exposure over time during each experiment. We have not attempted to equate a specific set of events with a specific remifentanil concentration, but we have considered testing time-points from 5 through 12.5 min to correspond to high opioid exposure based on the frequency of respiratory outcomes during quiet conditions. Although estimated opioid concentrations differed from subject to subject, we maintain that relative increase and subsequent decrease in concentration for each subject occurred in an incremental fashion. Second, the brevity of remifentanil infusion and rapid drug clearance limited the rise of CO_2_, blunting its potential to signal respiratory impairment, since hypercarbia requires time to manifest after immediate decline in minute ventilation. Third, although PUAL decline reflects opioid intensity, low PUAL does not imply absence of pain. For example, PUAL is fundamentally distinct from the variation coefficient of pupil diameter (CVPD), a measure recently cited as having value in identifying postoperative patients with higher pain scores [31] Finally, extrapolation of findings from volunteer subjects to patient populations should be regarded with caution. To determine whether pain, comorbid conditions, advanced age, or the concomitant use of centrally-acting medications alter the relationship between PUAL and opioid effect, ongoing studies of PUAL in diverse clinical populations will be needed.

Despite these limitations, we believe the implications of our findings will be relevant to clinicians in the future. Low or absent PUAL identifies individuals at risk for deterioration warranting additional precautions, especially in circumstances where environmental or nociceptive stimulation might be abruptly withdrawn.

## Data Availability

Pupillary data (diameter versus time) are available in csv files downloaded from the Neuroptics pupillometer. Other physiologic data are available in an Excel format.
